# Measurement of Adhesion of Sternal Wires to a Novel Bioactive Glass-Based Adhesive

**DOI:** 10.3390/jfb10030037

**Published:** 2019-08-09

**Authors:** Varinder Pal Singh Sidhu, Mark R. Towler, Marcello Papini

**Affiliations:** 1 Department of Mechanical & Industrial Engineering, Ryerson University, Toronto, ON M5B 2K3, Canada; 2 Li Ka Shing Knowledge Institute, St. Michael’s Hospital, Toronto, ON M5B 1A6, Canada

**Keywords:** glass polyalkenoate cement, sternal fixation, adhesive, bone cement, bioactive glass, stainless steel wire, resternotomy, friction, mechanical interlocking

## Abstract

Stainless steel wires are the standard method for sternal closure because of their strength and rigidity, the simplicity of the process, and the short healing time that results from their application. Despite this, problems still exist with sternal stability due to micromotion between the two halves of the dissected and wired sternum. Recently, a novel glass-based adhesive was developed which, in cadaveric trials and in conjunction with wiring, was shown to restrict this micromotion. However, in order to avoid complications during resternotomy, the adhesive should adhere only to the bone and not the sternal wire. In this study, sternal wires were embedded in 8 mm discs manufactured from the novel glass-based adhesive and the constructs were then incubated at 37 °C for one, seven, and 30 days. The discs were manufactured in two different thicknesses: 2 and 3 mm. Wire pull-out tests were then performed on the constructs at three different strain rates (1, 0.1, and 0.01 mm/min). No statistically significant difference in pull-out force was found regardless of incubation time, loading rate, or construct thickness. The pull-out forces recorded were consistent with static friction between the wire and adhesive, rather than the adhesion between them. Scanning electron micrographs provided further proof of this. These results indicate that the novel adhesive may be suitable for sternal fixation without complicating a potential resternotomy.

## 1. Introduction

Median sternotomy was introduced in the 1950s and is still the gold standard technique for providing access to the heart and lungs, which are protected by thorax structures [1,2]. A vertical incision is made along the length of the sternum for surgical procedures such as heart transplants, artery bypass surgeries, etc. The median sternotomy process begins with the separation of superficial sternal tissues. Following this, a longitudinal bisection/incision along the center of the sternum is made using a high-frequency saw. The bisected halves of the sternum are then held by the sternal retractors, facilitating the formation of an opening into the thoracic cavity. Following the surgical procedure, the surgeon reconnects the sternal halves using one of the techniques (wiring or plate screw system) for sternal closure. Sternal closure has a significant role in minimizing the complications after any thoracic operation [1,3,4].

Sternal instability occurs in a small but significant number (1 to 8%) of patients worldwide [5,6]. It is defined as the abnormal movement of the sternum because of a bony fracture of the sternum, micromotion between the two halves of the sternum after sternotomy, or disruption of the sternal wires introduced to rejoin the divided sternum. The consequences include pain and impaired function that may progress if not diagnosed prior to sternal infection and dehiscence [5,7,8,9]. Risk factors for sternal instability include pathologic conditions that affect bone healing such as obesity, diabetes, and COPD (Chronic Obstructive Pulmonary Disease); gender (female); and the occurrence of extensive sternal devascularization following resternotomy or bilateral mammary artery grafting [10,11].

Alhalawani and Towler reviewed the pros and cons of sternal fixation methods following median sternotomy [12]. Every year in the United States, nearly 7.5 million cardiac operations are performed, resulting in total healthcare costs of $315B [13]. Since 1957, stainless steel wires have been the standard method for sternal closure because of their strength and rigidity, the simplicity of the process, and the resultant short healing time [14]. However, wiring can lead to a lack of stability, resulting in micromotion between the two halves of the sternum [15]. An alternative to wire cerclage which has been proven to reduce sternal instability is rigid plate fixation (RPF) [16]; however, its high cost has restricted the technology from becoming broadly adopted. Moreover, there are inherent risks to the heart by drilling into the sternum [17] to fix the RPF devices and there is potential for disrupting sternal blood supply [18]. The use of two other sternal reinforcement devices, i.e., the Sternal Synthesis device (SSD) (Mikai SpA, Vicenza, Italy) and the Sternal Talon device (KLS Martin Group, Jacksonville, FL, USA), was proposed by Zeitani et al. [19] and Levin et al. [20], respectively. In comparison with wiring techniques, interlocking sternal fixation devices were found to provide better sternal fixation. However, these systems need long-term follow-up [12].

Deep sternal wound infection (DSWI) can arise in 0.3–5% cases and has been related to a mortality rate of up to 47%. The impact of sternal infection on the healthcare system is critical, almost tripling the cost of surgery [21]. Apart from DSWI, other complications include sternal dehiscence [22], difficulty in respiration [23], continuing pain [24], and mediastinitis [25], which are worsened by obesity and diabetes. 

An adhesive approach to sternal fixation has been postulated to reduce micromotion between the sternal halves which can result in postoperative pain, retarded sternal healing, and reduced quality of life [26]. Doctors Research Group (DRG, Southbury, CT, USA) developed Kryptonite™ bone cement. In 2009, DRG received 510(k) approvals from the US Food and Drug Administration (FDA) to market Kryptonite™ for cranioplasty applications. Under an investigational device exemption (IDE), it was used in combination with stainless steel wires to improve fixation in median sternotomy surgery. Kryptonite™ is a castor oil-derived adhesive, which was reported to prevent pathological sternal displacement and to have favorable biocompatibility and adherence to bone [27]. The clinical use of Kryptonite, under the IDE, was reported to prevent sternal dehiscence, thus decreasing postoperative pain [26]. However, during curing it experienced volumetric enlargement and it lacked the ability to impart an antibacterial effect to minimize DSWI [28]. Kryptonite™ is no longer available on the surgical market. However, its potential outlined by the IDE trials indicated the potential of an adhesive-based solution for sternal fixation.

In 1971, glass poly(alkenoate) cements (GPCs) were developed as dental restorative materials by Wilson and Kent [29]. GPCs can chemically adhere to bone [30] and be formulated to release ions such as zinc (Zn) and calcium (Ca) [31]. All commercial GPCs contain Al in the glass phase [32], which restricts their use for orthopedic applications because the release of Al ions has been associated with neurotoxic effects and degenerative brain diseases [33]. Investigators [34] have replaced Al with zinc (Zn) and strontium (Sr) in order to develop an Al-free version of the ionomeric glass. Zn has a positive result on bone metabolism [35] as it has antibacterial and anti-inflammatory properties [36,37]. Sr has the ability to replicate pre-osteoblastic cells [38] and simulate bone formation [39]. Mehrvar et al. performed cadaveric tests using such GPCs in conjunction with 316L stainless steel surgical wire, and found that they could provide immediate bone stability, significantly reducing sternal displacement. No adhesive-enhanced sternum experienced a pathological sternal displacement up to 500 N [40].

Resternotomy (RS) is a repeat sternotomy that is sometimes necessary following the prior sternotomy. A saw is used to reopen the full length of the sternum [41]. Every year, the number of patients undergoing RS continues to rise [42,43]. Compared to a primary cardiac operation, RS can be more technically challenging, with the potential for significant injury [42]. To perform RS, specialized instrumentation or techniques should be used to facilitate sternal re-entry or dissection and, in the case of sternal adhesives, RS is only possible if the adhesive does not bond to the metal wires [44]. Although initial sternotomy is not typically hazardous, the potential ensuing adhesion between the sternum adhesive and metal wires increases the potential for complications during sternal re-entry [45]. The removal of sternal wires can be an issue in the case of deep sternal wound infection (DSWI), the occurrence of which can be reduced by the incorporation of an antibacterial into the bioactive adhesive [46]. Moreover, the adhesive can fill the voids that potentially allow bacterial ingress, thus further reducing the potential for DSWI. Having a wire that does not adhere to the bioactive adhesive facilitates the removal of the wires, thus simplifying resternotomy in the presence of the adhesive. The aim of this study was to determine the extent of adhesion between 316L stainless steel surgical wire and the novel GPC adhesive which was used by Mehrvar et al. [40] to reduce sternal displacement. 

## 2. Materials and Methods

### 2.1. Glass and Adhesive Preparation

A glass ionomer cement (GIC) based on a proprietary SiO_2_-ZnO-CaO-SrO-P_2_O_5_-Ta_2_O_5_ glass was used in this study. The glass was prepared by a glass manufacturer (Mo-Sci, Rolla, MO, USA) using specified mole fractions and particle sizes (Table 1). This glass was then annealed at 670 °C for 12 h to relieve internal stresses within the glass network. The furnace (ZIRCAR Ceramics, Florida, NY, USA) was programmed to reach to the annealing temperature in 3 h and to cool down to the room temperature (25 ± 2 °C) in 3 h. These annealed glass powders were again sieved through 45 μm mesh.

The adhesive was prepared by weighing out the glass and polyacrylic acid (PAA) (Mw ~35,000 and median particle size <90 μm, Advanced Healthcare Ltd., Tonbridge, UK), and mixing with tri-sodium citrate (TSC) and water in a beaker in the proportions shown in Table 1, mixing thoroughly for 30 s.

### 2.2. Specimen Preparation

Wire pull-out testing specimens consisting of adhesive discs cast against centrally placed wires passing through the specimen thickness were made. In total, 90 adhesive samples were made, and arranged into two groups (Table 2) with adhesive disc thicknesses of 2 mm (45 samples) and 3 mm (45 samples). For these samples, 316L stainless steel surgical grade (Ethicon, USA) monofilament strands/wires of 1 mm diameter were used. The wires were first cleaned using acetone, and then held from one end by potting them in a cylindrical mold filled with AlumiRes RC-3 casting resin (Alumilite Corporation, Galesburg, MI, USA), which was allowed to cure for 10 h. The Alumilite cylinder facilitated the holding of the wire while fabricating and incubating the samples.

**Figure 1:**
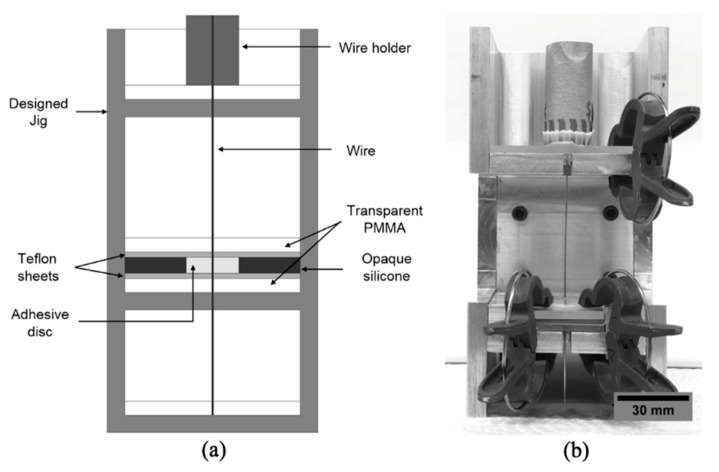
(**a**) Design of the jig used for specimen preparation; (**b**) image of the jig.

To cast the adhesive with the inserted wire, 8 mm circular silicon molds with thicknesses of 2 and 3 mm were used. The silicon molds were made using a hammer-driven hole punch (Mcmaster-Carr, Elmhurst, IL, USA). Each silicon mold was placed in between PMMA (Poly(methyl methacrylate)) plates of the same dimensions with a circular hole in the center on the bottom PMMA plate, and a slot in the center on the upper PMMA plate. A Teflon film of thickness 0.127 mm with a hole in the center (with the same dimensions of PMMA and mold) was placed between the silicon mold and the PMMA. A jig was designed to ensure that the wire–adhesive disc specimens were aligned, as shown in Figure 1. The Teflon sheet, PMMA, and silicon mold were placed in the jig and the wire was inserted into it. The prepared adhesive was then poured into the mold. Soft clips were used to stop any minute movement of apparatus (PMMA, mold, wire) as well as to hold the apparatus precisely. 

**Figure 2:**
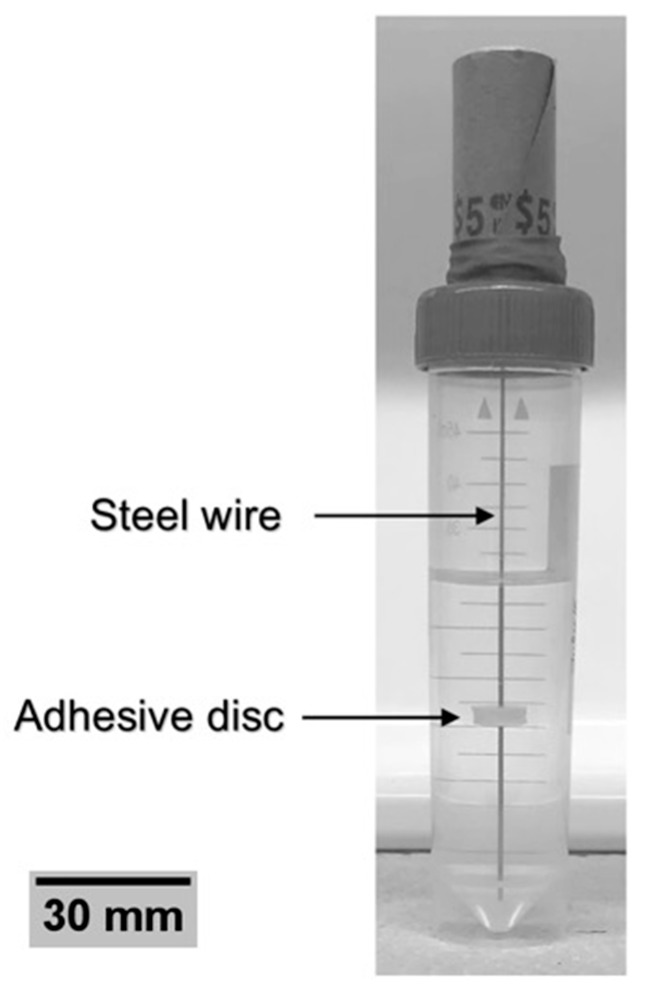
Specimen incubation in deionized water.

The apparatus was inserted into an oven at 37 °C and the adhesive was dried for 1 h. The specimen was then demolded from the silicone, leaving the adhesive disc in contact with the wire. In order to assess the effect of incubation on the adhesion with the wire, the specimens were inserted into deionized water and placed into an incubator at 37 °C (Figure 2). A total of n = 15 samples of each disc thickness were incubated for 1, 7, and 30 days. 

### 2.3. Wire Pull-Out Tests

**Figure 3:**
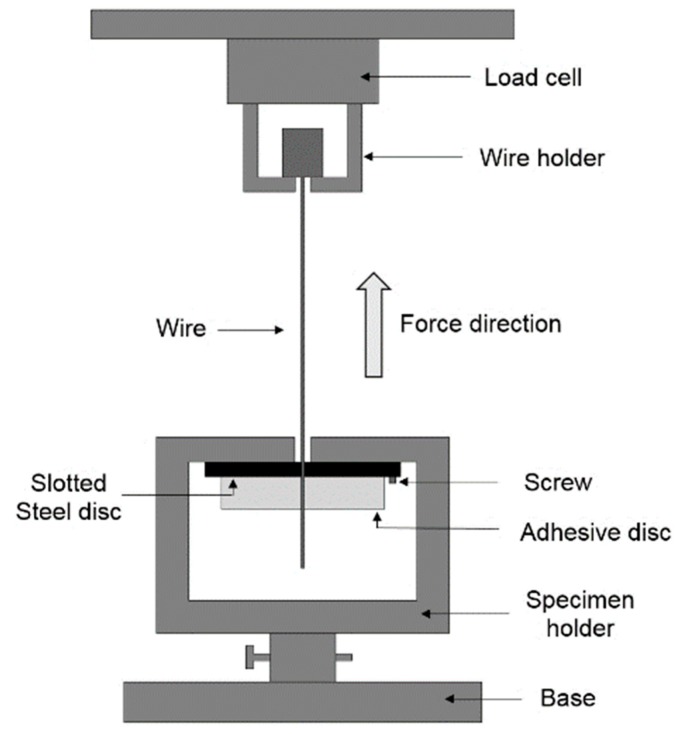
Wire pull-out test apparatus. (Figure is not to scale; i.e., the adhesive disc is shown much larger for clarity).

After the required incubation time, pull-out tests were performed using a tensile testing system with a load cell of 5 kN and an accuracy of ±0.15 N (United Testing Systems Inc., Fullerton, CA, USA). As in previous studies that have utilized pull-out tests to assess adhesion between steel wires and adhesive resin [47], displacement rates of 1, 0.1, and 0.01 mm/min were used. The setup used for the pull-out test (shown in Figure 3) is similar to that previously used in [48,49] to test the adhesion of orthodontic adhesives to wires and to study the tensile and fracture properties of acrylic bone cements reinforced with both silane-coated and uncoated 316L stainless steel fibers. The adhesive–wire sample was placed in the wire and specimen holders as shown in Figure 3. The holders ensured that the wire was vertical and directly in line with the load cell and the direction of force application. A slotted circular steel disc (with a slot located 2 mm from the center) was screwed to the specimen holder. The force and crosshead displacement were recorded during the test and used to report the maximum force prior to wire pull-out.

### 2.4. Statistical Analysis

The pull-out forces were reported as the mean ± standard error for n = 5 samples. The analysis of variance (ANOVA) test was used to assess the statistical significance of the data. The independent samples t-test with p < 0.05 was used in a post-hoc analysis to determine the statistical significance of the effect of incubation days, adhesive thickness, and loading rates.

## 3. Results and Discussion

### 3.1. Force–Displacement Curves and Likely Mechanism for Force Generation

**Figure 4:**
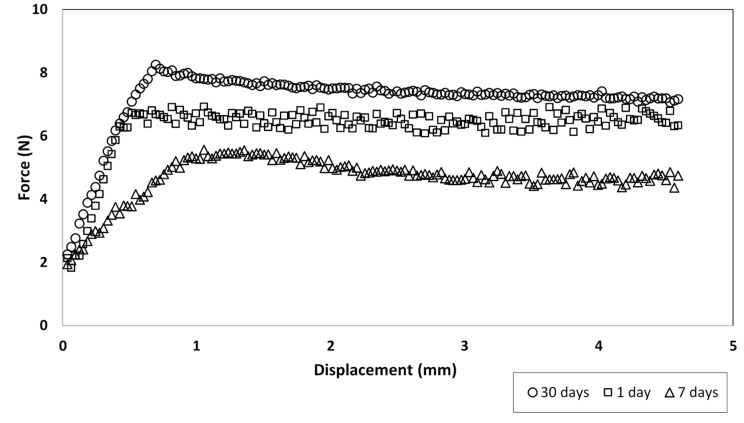
Force–displacement curves of 3 mm adhesive disc at strain rate of 1 mm/min.

The typical force–displacement curve shown in Figure 4 illustrates that the force typically plateaued at a relatively low value after a certain amount of displacement. The absence of a sharp drop in force after the peak was reached indicates that the wires did not adhere as the pull-out began. The same trend was noticed by Mann et al. during pull-out tests of Ti alloy stems and poly(methyl methacrylate) bone cement [50]. They attributed this behavior to an absence of chemical adhesion, where the load–displacement behavior could be described completely in terms of the friction at the interface. Therefore, the curve in Figure 4 is consistent with the presence of friction between the adhesive and wire as it was pulled out, rather than a loss of adhesion. Since the general shape of the curve and behavior of all the tested samples were similar, Figure 4 plots the case of a 3 mm thick disc which was pulled out at a strain rate of 1 mm/min after 1, 7, and 30 days of incubation, as a representative.

### 3.2. Effect of Incubation

**Figure 5:**
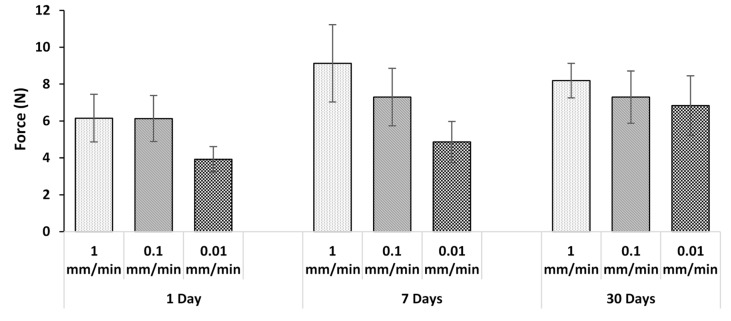
Average force with 2 mm adhesive disc after pull-out test.

**Figure 6:**
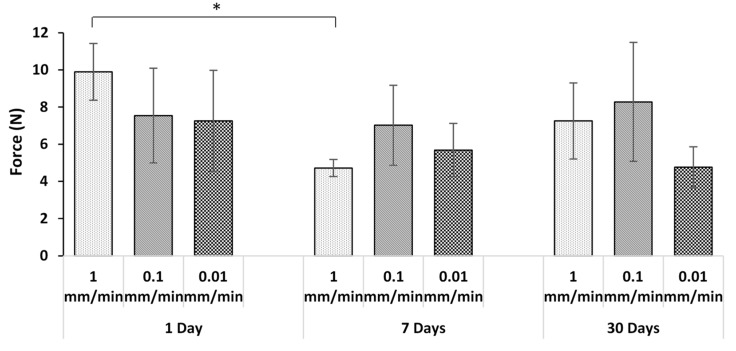
Average force with 3 mm adhesive disc after pull-out test (the asterisk and bar show statistical significance at p < 0.05).

**Figure 7:**
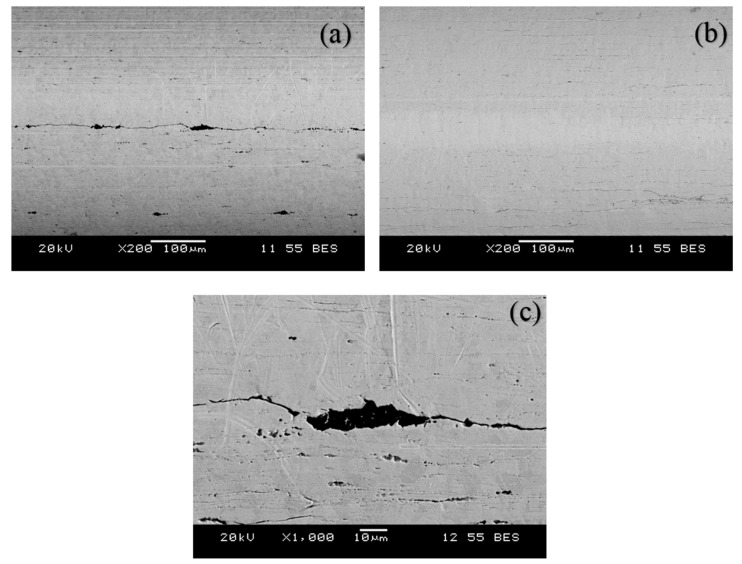
Scanning electron microscopy images of wire surfaces after the pull-out test of same group. (**a**) Wire sample with high pull-out force; (**b**) wire sample with low pull-out force; (**c**) enlargement of the box in (**a**) showing adhesive entrapped in the voids.

Figure 5 and Figure 6 compare the pull-out forces after three different incubation times for the two sample thicknesses tested at various strain rates. With the exception of one case, at a given displacement rate, and for both thicknesses, there was no statistically significant effect of incubation time on the pull-out force. The absence of dependence on incubation time again points to a lack of adhesion. The only exception was for the 3 mm thick sample (Figure 6) at 1 mm/min after 1 and 7 days incubation. This case is clearly an outlier, likely due to variations in the surface topography of the wire which affect the degree of mechanical interlocking. For example, voids can be seen on the wire surface in Figure 7. This will be discussed further in Section 3.5. 

### 3.3. Effect of Displacement Rate

At any given incubation time, there was no statistically significant effect of displacement rate on the pull-out force for both specimen thicknesses. This was expected since, as the previous sections have implied, the pull-out force was largely due to friction between the adhesive disc and wire rather than adhesion, and the laws of dry friction imply that the kinetic friction force does not change with sliding velocity [51]. 

### 3.4. Effect of Thickness

For any given displacement rate and incubation time, there was no statistically significant effect of the thickness of adhesive disc on the pull-out load, except for samples with 1 day incubation at 1 mm/min. As already discussed in Section 3.2, this difference was likely due to differences in wire surface texture causing different degrees of mechanical interlocking. This was again expected given the frictional mechanism for pull-out, since friction is largely independent of the contact area [52]. 

### 3.5. Mechanism of Generation of Pull-Out Force

As shown by the results discussed above, the most likely mechanism for the generation of the pull-out force was friction, rather than chemical adhesion. Adhesion is not solely a result of ion bridging, but of interactions between the polyacrylate chains and the surface. In other words, adhesion is dynamic in nature with bond interchange and ion exchange between the adhesive interface and substrate surface. There cannot be any ion transfer from the metal wire at a normal temperature. Thus, adhesion can only occur when an adhesive and a substrate are brought into molecular contact. In this adhesive, PAA is responsible for adhesion to bone because its functional group has the ability to form a multiplicity of hydrogen bonds [41]. However, the chromium in 316L stainless steel does not allow the OH group of PAA to react with 316L stainless steel. There is neither an ion transfer nor molecular bond formation between PAA and 316L stainless steel, and therefore it is not surprising that there was no adhesion.

Scanning electron microscopy (SEM) of the wires was performed after the pull-out test to determine if there were any adhesive particles on the surface of the wire. Two samples were chosen, one with the highest force and the other with the lowest force in the same group of five samples. Thus, a total of 36 samples of 18 groups were scanned by SEM. It was found that the wire surfaces were virtually void of adhesive (Figure 7), confirming that if any failure occurred, it was interfacial along the wire surface. The low-magnitude pull-out forces, together with the trends discussed in Section 3.1, Section 3.2, Section 3.3 and Section 3.4, indicate that the behavior that was more consistent with a frictional mechanism of force generation. 

Although chemical adhesion did not occur, it is likely that some degree of mechanical interlocking occurred due to the ingress of adhesive into small voids that were present on the wire surface. With the exception of one group, for all other groups of five samples with the same displacement rate, thickness, and incubation time, no more than one sample had a pull-out force higher than 10 N. The exception was the group of 3 mm discs that were incubated for 1 day and tested at 1 mm/min, in which three samples had pull-out forces >10 N. This corresponds to the statistically significant outlier in Figure 6. Figure 7 shows the SEM images of two samples within that group, corresponding to the highest (13.4 N) and lowest (5.6 N) forces. It can be seen that Figure 7a (higher load) has more cracks and voids than Figure 7b, which is smoother. It was thus assumed that the higher force is due to the mechanical interlocking of adhesive into the microvoids/microcracks on the wire. 

**Figure 8:**
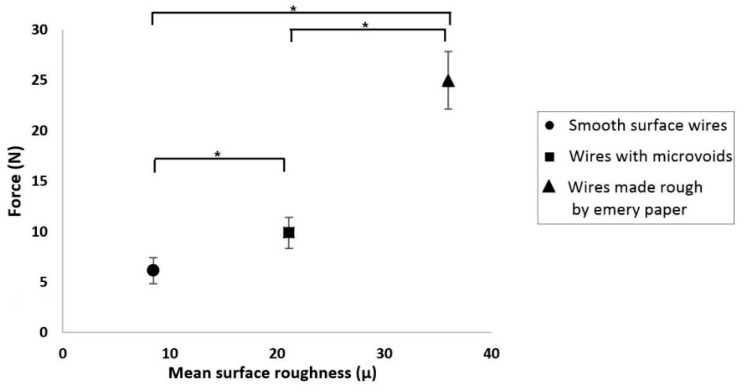
Force vs. mean surface roughness of wire (the asterisks and bars show statistical significance at p < 0.05).

In order to further investigate the effect of the wire roughness on mechanical interlocking, a further five samples of 3 mm thickness were made. The same 316L stainless steel surgical wire was used, but this time the surface of the wire was made rough using P120 emery paper with an average grit size of 125 μm. The roughness of the wire surface was measured using a non-contact optical profilometer (NANOVEA ST400, Irvine, CA, USA). Samples were prepared in the manner described in Section 2.2 and incubated for 1 day. Figure 8 shows the effect of surface roughness on the pull-out force for smooth wires (Figure 7b), wires with microvoids (Figure 7a; wires from 3 mm thick samples with pull-out forces >10 N for discs incubated for 1 day and tested at 1 mm/min), and the roughened wires. There were significant differences among the pull-out force of the smooth wires, wires with microvoids, and wires made rough using emery paper. 

The average pull-out force for these rough wire samples was 25 N with a standard error of ±2.86 N, i.e., over twice as high as the forces using non-roughened, as-received wires (Figure 6).

It is thus clear that the mechanical interlocking of adhesive increases with high surface roughness. As such, the group that showed statistically significant differences (three wire samples of 3 mm that were incubated for 1 day and then tested at 1 mm/min, having pull-out forces >10 N, as shown in Figure 7a), was due to the interlocking of the adhesive into the microvoids/microcracks on the wire surface. This mechanical interlocking can be decreased by using wires with smooth surfaces. However, overall the measured pull-out forces were consistent with static friction between the wire and adhesive, rather than adhesion between them.

## 4. Conclusions

The results of the pull-out tests used to assess the adhesion between a glass-based adhesive and a sternal wire revealed that:

1. In all but one case, there was no statistically significant effect of adhesive thickness, loading rate, or incubation time on the pull-out forces. In the outlying case, it was demonstrated that the differences were due to variable wire surface roughness.
2. The pull-out forces were due to friction between the adhesive and wire, rather than chemical adhesion.
3. The variation of forces within the samples was due to slight differences in the mechanical interlocking of the adhesive into microvoids that existed on some of the wires. As expected, smoother wires generated less friction.

This work is the first to assess the adhesion of a novel bioglass adhesive to 316L stainless steel surgical wires. The results show that, when used with sternal closure wires, the adhesive is not likely to present difficulties associated with bonded wires during resterotomy procedures.

## Figures and Tables

**Table 1:** Composition of glass, particle size, and adhesive formulations. PAA = polyacrylic acid; TSC = tri-sodium citrate.

Glass Composition (Mole Percentage)						Glass Particle Size (μm)	Adhesive Formulation
SiO_2_	ZnO	CaO	SrO	P_2_O_5_	Ta_2_O_5_		Glass (g):PAA (g):Water (mL):TSC (g)
48	35.5	6	8	2	0.5	<45	1:0.4:0.6:0.075

**Table 2:** Experimental conditions.

Adhesive Disc Thickness	2 mm									3 mm								
**Incubation Days**	1			7			30			1			7			30		
**Loading Rates (mm/min)**	1	0.1	0.01	1	0.1	0.01	1	0.1	0.01	1	0.1	0.01	1	0.1	0.01	1	0.1	0.01
